# The Paths to Connectedness: A Review of the Antecedents of Connectedness to Nature

**DOI:** 10.3389/fpsyg.2021.763231

**Published:** 2021-11-04

**Authors:** Michael L. Lengieza, Janet K. Swim

**Affiliations:** Department of Psychology, The Pennsylvania State University, University Park, PA, United States

**Keywords:** connectedness to nature, contact with nature, self, antecedents, literature review

## Abstract

Although many philosophers and environmental psychologists agree that progress toward a more ecologically conscious society depends upon individuals developing a sense of connectedness to nature, such agreement is of limited use if we do not understand how connectedness forms. The purpose of this review is to delineate the state of the psychological literature concerning the antecedents of connectedness to nature. The literature review is organized into three main sections: (1) situational contexts that influence connectedness; (2) individual difference predictors, such as demographic group membership, personality, or beliefs; and (3) internal psychological states that may explain psychological processes that result in connectedness. Major critiques of the extant literature and future directions are presented in a discussion following the body of the review. The primary implications highlighted by the review are a greater need for theories delineating the formation of connectedness, a greater focus on process, and increased differentiation between similar antecedents of connectedness.

## Introduction

Philosophers, environmentalists, and psychologists alike contend that a critical step in the progression toward a more environmentally responsible society is coming to include nature within our sphere of concern. That is, people must make nature a part of what or who they deem important enough to be deserving of concern and protection (Leopold, 1949; Naess, 1987; Stern et al., 1999; Schultz, 2002; Crimston et al., 2016). For Leopold (1949), this meant including nature in our ethical frameworks, like we would a traditional community member. Naess (1987), in particular, goes further by noting the value of including nature in our self, and our self in nature, a view notably consistent with that adopted by psychologists (e.g., Schultz, 2002). Supporting the contentions of philosophers and psychologists, individuals who feel more connected to nature—that is, who include nature in their sense of self—are more pro-environmentally disposed (e.g., Davis et al., 2011) and tend to have better psychological wellbeing (e.g., Mayer et al., 2009). Thus, connectedness to nature is of particular interest because it may simultaneously promote the health of both the planet and people.

The purpose of this review is to delineate the state of the psychological literature concerning the antecedents of connectedness to nature in hopes of moving the field toward the development of theory. Although philosophers and environmental psychologists agree upon the importance of connectedness, such agreement is of limited use if we do not understand how a sense of connectedness forms and, consequently, can be fostered. Thus, a call for increased connectedness to nature begs the question of how this sense of connection might develop. We first define connectedness to nature. Then, we describe the literature review and findings. Last, we consider implications of the reviewed findings for areas future research.

### Definitions of Connectedness to Nature

Definitions of connectedness to nature (connectedness hereafter) found in the psychological literature emphasize a merging of self and nature (Schultz, 2002) and a sense of oneness or unity with nature (Mayer and Frantz, 2004). Both the merging of self and nature and the sense of oneness align with individuals’ explanation of what connectedness means to them (Unsworth et al., 2016). These two points of emphasis are also consistent with viewing connectedness as a form of self-transcendence (Lengieza et al., 2021). Specifically, self-transcendence is characterized by “decreased salience of the self, accompanied by a softening or complete dissolution of the conceptual boundaries between self and others, involving a sense of oneness with others and one’s surroundings” (Lengieza et al., 2021, p. 5; see also Yaden et al., 2017). Given these considerations, we define connectedness as psychological joining of nature and the self which manifests as a sense of oneness with nature.^1^

## Literature Review

The purpose of the review is to identify the general state of the psychological literature on the causes of connectedness to identify the key concepts that predict and help explain the development of connectedness and provide future directions for research. While thorough, the review is not meant to be exhaustive but instead is meant to capture the most prominent trends in the psychological literature on connectedness to nature.

### Method

First, a search of peer-reviewed empirical articles in PsychINFO and PsychArticles, conducted *via* ProQuest was used to focus on empirical studies that would provide evidence of psychological predictors of connectedness. The search terms used were: “connectedness,” “connection to nature,” “connectedness with nature,” “connection with nature,” “nature relatedness,” “nature connectedness,” “environmental identity,” “Inclusion of nature in the self,” “Inclusion of nature in self,” “nature connectivity,” “connectivity to nature,” “connectivity with nature,” “disposition to connect with nature,” “disposition to connect to nature,” “emotional affinity to nature,” “emotional affinity with nature,” and “love and care for nature” appearing anywhere in the abstract up to and including the year 2019. These search terms were informed by the first author’s experience with the literature and, more specifically, the constructs included in Tam (2013) paper investigating the overlap between constructs used to study connectedness. The breadth of search terms was selected to ensure that relevant articles were not blindly excluded from the review. The noted search terms returned 323 articles.

The first author read the abstracts of the 323 articles to determine which articles warranted further reading. First, papers that did not mention connectedness as an observed construct or made it clear that they measured environmental*ist* identity were excluded (*n*=170) reducing the number of articles to 153. Second, articles were *pragmatically* excluded—based upon the abstract—based on treating connectedness as predictor, and not an outcome were excluded (*n*=68), which reduced the number of articles to 85.^2^ It is important to note that, since this step was motivated by pragmatism, sources that the first author had previously read and were already known to be relevant to the review but otherwise would have been excluded at this step were ultimately included in the narrowed pool referenced below. The articles from the narrowed pool (*N*=85) were then read to determine the general themes in the literature. Table 1 presents the sources included in the review and in which of the sections they appear. The table also notes the subthemes that are covered in detail in the qualitative review of these studies.

**Table 1:** Sources included in the review and the location of their appearances.

Citation by paper	Year	Study	Situational contexts			Individual differences			Psychological factors		
			Contact	Mediat.	Activ.	Demo.	Pers.	World.	Mindf.	Self	Affect
Ahn et al.	2016	1		X						X	
		2		X						X	
		3		X						X	
Aspy and Proeve	2017	1			X						
Barbaro and Pickett	2016	1							X		
		2							X		
Barton et al.	2016	1	X			X					
Beery	2013	1	X		X	X					
Braun and Dierkes	2017	1	X		X	X					
Brick and Lewis	2014	1					X	X			
Brown	2017	1	X			X		X			
Bruni and Schultz	2010	1	X			X		X			
		2	X			X		X			
		3	X		X	X		X			
Bruni et al.	2008	1	X			X					
Bruni et al.	2017	1			X						
		2	X								
		3		X							
Burbach et al.	2012	1	X		X	X					
Capaldi et al.	2014	1				X					X
Capaldi et al.	2017	1						X			X
		2						X			X
Cheng and Monroe	2012	1	X					X			
Cho and Lee	2018	1			X						
Clayton et al.	2011	1	X		X			X			
Crawford et al.	2017	1			X	X		X			X
Crimston et al.	2016	3						X			
Davis and Stroink	2016a	1				X		X			
Davis and Stroink	2016b	1				X		X		X	
Davis et al.	2011	1						X			
DiFabio and Bucci	2016	1					X	X			
Di Fabio and Kenny	2018	1					X	X			
Diessner et al.	2018	1				X		X			
Dopko et al.	2019	1			X						X
Dopko et al.	2014	1		X							X
		2		X							
Duffy and Verges	2010	1	X					X			
Dutcher et al.	2007	1				X		X			
Ernst and Theimer	2011	1			X						
Forstmann and Sagioglou	2017	1			X		X				
Frantz et al.	2005	1				X		X		X	
		2				X		X		X	
Hanley et al.	2017	1							X		
Hanley et al.	2016	1	X			X					
Hedlund-de Witt	2014	1						X			X
Hinds and Sparks	2009	1	X								X
Howell et al.	2011	1							X		X
		2							X		X
Hughes et al.	2019	1				X					
Johnson-Pynn et al.	2014	1			X						
Kals et al.	1999	1	X								
Lankenau	2018	1			X						
Larson et al.	2018	1	X			X					
Lee et al.	2015	1					X	X			
Lengieza and Swim	2021	1	X							X	
Lengieza et al.	2021	1			X						X
Liefländer et al.	2013	1				X					
		2			X	X					
Liu et al.	2019	3			X	X					
Lumber et al.	2017	1	X					X			
		2	X					X			
		3	X					X			
Lyons and Carhart-Harris	2018	1			X						
Mayer and Frantz	2004	1	X			X		X			
		2				X		X			
		3			X						
		4						X			
		5						X			
Mayer et al.	2009	1	X			X				X	X
		2	X	X		X				X	X
		3	X	X		X				X	X
Nisbet and Zelenski	2011	1	X								X
		2	X								X
Nisbet et al.	2019	1	X		X						X
Nisbet et al.	2009	1	X				X	X			
		2	X				X				
Nisbet et al.	2011	1									X
		2									X
		3									X
Nour et al.	2017	1			X	X	X	X		X	
Otto and Pensini	2017	1			X						
Passmore and Holder	2017	1			X						
Pensini et al.	2016	2	X			X					
Poon et al.	2015	1									X
		3									X
Richardson and Sheffield	2015	1							X	X	
		2					X		X	X	
		3		X					X	X	
Richardson et al.	2016	1	X								X
Rosa et al.	2018	1	X								
Sanguinetti	2014	1			X	X		X			
Schultz and Tabanico	2007	1						X			
		2	X					X			
		3	X			X					
		4	X			X					
		5	X								X
Schutte and Malouff	2018	1				X			X		
Scott	2010	1	X					X		X	
		2	X					X		X	
		3		X						X	
Sellmann and Bogner	2013	1			X			X			
Soliman et al.	2017	1		X							
Spendrup et al.	2016	1	X			X					
Swami et al.	2016	1	X			X				X	
Tam	2013	1					X	X			
		2	X								
Tam et al.	2013	2			X						
		3			X						
Unsworth et al.	2016	1				X			X		
		2	X		X	X			X		
Vess et al.	2012	1						X			
		2						X			
		3						X			
Walters et al.	2014	1	X			X					
Wang et al.	2019	1			X						
Wang et al.	2016	1			X						
Weinstein et al.	2009	2		X		X					
		3		X		X					
		4	X			X					
Wheaton et al.	2016	1	X								
Whitburn et al.	2019	1			X	X		X			
Wyles et al.	2019	1	X		X	X					
Yang et al.	2018	2									X
		3									X
Zelenski et al.	2015	1		X							
		3		X							
Zhang et al.	2014b	1				X	X	X			
		2				X		X		X	
Column totals			47	15	32	46	11	45	11	19	36

## Findings

The body of the review is organized according to the three emergent themes identified in the literature: (1) situational contexts associated with connectedness; (2) individual difference predictors, such as demographic group membership, personality, and beliefs; and (3) internal psychological states that may explain psychological processes that result in. Each section ends with a summary of the findings outlined in that section. After detailing findings from the review, in the discussion, we highlight gaps and future directions for the study of the antecedents of connectedness that emerge when considering the body of literature as a whole. Table 2 presents the number of studies appearing in each section broken down by relevant attributes.

**Table 2:** Counts of studies appearing in each section broken out by relevant attributes.

Section	Total	Measure						Gender composition^1^			Sample age		
		INS	CNS	NR	IAT	Combo	Other	≥55% W	Equal	≥55% M	Child	College	Other
Situational	84	18	20	9	9	14	13	46	20	7	15	36	32
Individual	72	10	29	8	6	9	10	44	19	5	10	30	32
Psychological	44	10	19	4	1	4	6	27	9	5	2	28	14

Total reflects the number of studies, not papers. Rows are not mutually exclusive; columns within an attribute category are.

1 Some studies did not report their sample gender composition.

The situational contexts section outlines the ways in which contact with nature, in a variety of forms and doing various activities during contact, promote or suppress connectedness. The individual differences section touches on the influence that demographic characteristics, personality, and worldviews have on connectedness. The psychological states section details how connectedness is impacted by psychological states related to mindfulness, the self, and affect.

### Situational Contexts That Influence Connectedness

Several of the known antecedents to connectedness can be broadly characterized as “situational contexts.” These situational contexts can involve (a) experiences with nature that occur through different mediums, such as contact with actual nature and contact with virtual nature, and can involve (b) different activities, such as participating in environmental education programs (e.g., Liefländer et al., 2013) or meditation (e.g., Nisbet et al., 2019). Knowing what kinds of situational contexts tend to result in connectedness reveals where and when connectedness tends to flourish and where and when it tends to struggle. These insights can both help to select ideal contexts upon which to focus efforts to promote connectedness and help identify other perhaps not-so-obvious, but theoretically relevant, contexts that may also increase connectedness. In other words, the type of research reviewed in this section will help identify where (in which contexts) and when (during which activities) to target efforts to promote connectedness.

#### Experiences With Nature

One of the most studied predictors of connectedness is contact with nature. The mediums of contact that have been studied range from actual, first-hand contact, most of which is in the form of spending time out in nature (e.g., Mayer et al., 2009), to mediated contact, often in the form of viewing pictures (e.g., Richardson and Sheffield, 2015) or watching videos of nature (e.g., Soliman et al., 2017), but increasingly includes more immersive experiences provided by virtual reality (e.g., Ahn et al., 2016).

##### Actual Contact With Nature

Many studies converge on the value of studying contact as a predictor of connectedness, demonstrating that contact (e.g., Kals et al., 1999; Mayer and Frantz, 2004, S1; Schultz and Tabanico, 2007, S3–5; Nisbet et al., 2009; Beery, 2013; Tam, 2013; Braun and Dierkes, 2017; Lumber et al., 2017), in a variety of forms, improves connectedness—whether it be contact with nature through nature-based tourism (Burbach et al., 2012; Wheaton et al., 2016) wilderness expeditions (Barton et al., 2016; Richardson et al., 2016), or contact *via* walking in nature (Mayer et al., 2009; Nisbet and Zelenski, 2011; Nisbet et al., 2019). In addition to contact with relatively organic forms of nature, contact with contrived nature, such as zoos, can foster a sense of connectedness (e.g., Schultz and Tabanico, 2007; Bruni et al., 2008). There is also substantial evidence that living closer to nature (e.g., Cheng and Monroe, 2012), living in a rural environment (e.g., Hinds and Sparks, 2009; Harvey et al., 2016) and more frequent exposure to nature (e.g., Kals et al.,1999; Mayer and Frantz, 2004, S1; Schultz and Tabanico, 2007, S5; Hinds and Sparks, 2009; Nisbet et al., 2009; Scott, 2010, S1–2; Tam, 2013, S2; Pensini et al., 2016; Richardson et al., 2016; Swami et al., 2016; Larson et al., 2018; Rosa et al., 2018) are associated with higher levels of connectedness. Contact with nature can even be as subtle as exposure to plants in a lab space (Weinstein et al., 2009). There is also some evidence to suggest that literal contact with nature may facilitate connectedness: one study found that comfort level walking barefoot is associated with increased connectedness; however, the causal direction of this relationship remains subject to interpretation and in need of further research (Harvey et al., 2016). Ultimately, many studies investigating the effect of contact with actual nature on connectedness conclude that it has a positive effect.

###### Characteristics of the Situation

The characteristics of the situation—the presence of certain attributes (e.g., greenery, water, etc.) as well as other situational elements of the nature experience (e.g., weather, immersion, etc.)—appear to influence the effect of contact on connectedness. Higher-quality natural areas (i.e., protected areas) are more effective in promoting connectedness than are lower-quality natural areas (Wyles et al., 2019). Additionally, rural green spaces seem to result in more connectedness compared to coastal blue spaces (e.g., oceans; Wyles et al., 2019). The importance of different features of nature on one’s sense of connectedness is consistent with research in biophilia which argues that people prefer environments that include features that improved human survival, including water (aka, the “blue effect”), landscapes that improve people’s ability to see long distances (prospect) or hide from predators (refuge; Dosen and Ostwald, 2016). Additionally, more global factors such as weather and season may influence connectedness. As the reader might suspect, connectedness is lower in the winter compared to autumn and spring and lower on rainy days compared to non-rainy days (Duffy and Verges, 2010). It may also be the case that variation in intensity of contact influences whether or not studies find an effect. For example, individuals report feeling more connectedness with longer contact with nature (Wyles et al., 2019). Further, the effects of exposure to plants on connectedness depends upon participants level of immersion. Participants who were immersed in a nature condition felt greater connectedness, whereas in a non-nature condition, the opposite was true (Weinstein et al., 2009), which would suggest that being more absorbed in natural environments facilitates connectedness, whereas being absorbed by non-nature environments may diminish connectedness.

The sizeable body of studies identifying contact with nature as a predictor of connectedness notwithstanding, a few studies fail to find an effect of contact with nature on connectedness (e.g., Bruni and Schultz, 2010; Clayton et al., 2011; Unsworth et al., 2016, S2; Bruni et al., 2017, S2; Lumber et al., 2017; Lengieza and Swim, 2021). For the most part, the reason for these null findings is unclear. On the one hand, the lack of effect may be attributable to mundane limitations, such as self-selection (e.g., Unsworth et al., 2016). Yet, the lack of effect may be meaningful. For example, studies may be more likely to find an effect when using comparison conditions that are more distinct from each other, such as comparing walking outside to walking inside (Nisbet and Zelenski, 2011), whereas those that use more similar conditions (dense vs. sparse nature, Lengieza and Swim, 2021) may not be as likely to detect an effect. If such a comparison were made within a study, rather than across separate studies, it might more readily reveal theoretically valuable insights about what *type* of contact is necessary to promote connectedness; in the case of this example, perhaps all that is important is having participants walk outside.

###### Childhood Contact With Nature

The above research focused on adult experiences in nature; however, several studies have also looked at the importance of childhood contact with nature. Like the trend noted above, these often-retrospective studies generally conclude that childhood contact with nature is a determinant of connectedness (Hinds and Sparks, 2009; Cheng and Monroe, 2012; Beery, 2013; Tam, 2013; Pensini et al., 2016; Rosa et al., 2018). However, some studies suggest that the primary avenue through which childhood contact exerts its influence on connectedness is through its influence on subsequent adult contact with nature (Pensini et al., 2016; Rosa et al., 2018). Still, much more can be learned about contact across the lifespan. Contact may be more potent at different stages in one’s life, perhaps being more integral to the development of the self at early stages in one’s life or being more impactful during transitional periods. What is more, some forms of contact might turn out to be more important than others at different periods of one’s life.

##### Mediated Contact With Nature

###### Pictures and Videos as Contact With Nature

Like the evidence that actual, first-hand contact with nature results in increased connectedness, several studies conclude that mediated contact with nature increases connectedness. Studies suggest that viewing pictures of nature (e.g., Scott, 2010, S3; Richardson and Sheffield, 2015) or videos of nature (Mayer et al., 2009, S2–3; Zelenski et al., 2015, S3; Soliman et al., 2017) can lead to increased connectedness, though, as with exposure to actual nature such as plants, this may depend on immersion (e.g., Weinstein et al., 2009; but also see Soliman et al., 2017).^3^ Though viewing nature in the form of videos and pictures has been identified as a predictor of connectedness, it is important to acknowledge that some studies report no effect of viewing pictures (Dopko et al., 2014, S1–2) or videos of nature (Zelenski et al., 2015, S1). Additionally, while pictures and videos of nature appear to promote connectedness, the effect of videos—and likely the effect of pictures as well—may fall short of spending time in actual nature (e.g., Mayer et al., 2009, S2–3).

###### Virtual Reality as Contact With Nature

Between full-fledged contact with actual nature and viewing videos or pictures of nature is exposure to nature *via* virtual reality (VR). Due to its infancy, a clear picture has yet to emerge from this line of research and there are not studies comparing VR to actual contact with nature. At present, however, two studies (i.e., Ahn et al., 2016, S1–2) demonstrate that VR is better than ordinary video. In contrast, two studies (i.e., Ahn et al., 2016, S3; Soliman et al., 2017) suggest that VR has no benefit (vs. video) and a third study with children suggests that levels of connectedness were no different before and after a virtual hike (Bruni et al., 2017, S3).

Infancy and ambiguity aside, the research on VR does point to one potentially valuable theoretical insight. Namely that one of the mechanisms through which VR may have its influence on connectedness is body transference (the perception of owning the body of the experienced avatar, something likely unique to VR; Ahn et al., 2016). This highlights that VR may operate through different mechanisms than other forms of contact with nature. Despite this interesting possibility, ultimately, the only conclusion to be drawn from the literature on VR at present is that we simply do not know whether VR consistently enhances connectedness.

##### Summary and Critique of Research on Contact With Nature

Contact with nature, both as child (e.g., Tam, 2013) and as an adult, is perhaps the most well documented antecedent of connectedness; research has consistently shown that spending time around or in nature (e.g., Mayer et al., 2009), viewing nature (e.g., videos; Richardson and Sheffield, 2015), or otherwise experiencing nature (e.g., VR; Ahn et al., 2016) can foster a sense of connectedness. Despite this consistency, there are several areas for advancement.

As noted above, while much research has been dedicated to understanding *if* contact with nature can increase connectedness, little, if any, research has adequately addressed the question of when and why contact with nature might not increase connectedness—cases where contact with nature may simply not have an effect, cases where some feature of the experience inhibits the normally positive effect, or, most importantly, cases where something about the situation actively *diminishes* connectedness. There are several studies in which contact with nature did not seem to affect connectedness (e.g., Lumber et al., 2017). Yet, we do not have a framework to understand why some studies find these null effects of contact with nature (e.g., Lengieza and Swim, 2021) in order to determine which null effects are theoretically meaningful and which are most likely methodological flukes. For example, one study investigated whether nature sounds would increase connectedness but found no evidence to support this notion (Spendrup et al., 2016). It might be easy to explain these findings by assuming that the manipulation may not have been sufficiently salient to be effective. However, to illustrate, these findings might have important theoretical implications; it may be that at a certain point, some contact is superficial or insufficiently meaningful to alter our mental landscape or, perhaps more importantly for theory, it may potentially be that nature sounds are not an important part of the effect of contact on connectedness. Ultimately, more attention should be paid to understanding when contact does not increase connectedness to disentangle methodological limitations from theoretically meaningful boundary conditions.

Moreover, we do not have a framework to predict when contact with nature should be expected to diminish connectedness. That is, there is virtually no research about conditions when contact with nature results in decreased connectedness. For example, contact with the “bugs and mud” of nature might create an aversive experience that counteracts the usually positive effect of nature, perhaps because such exposure feels threatening (see research on negative affect in later sections). The two studies appearing in this review closest to answering such questions investigated the how connectedness is affected by exposure to natural disasters—by all accounts a less than positive encounter with nature (e.g., Walters et al., 2014; Brown, 2017). While the results paint opposing pictures—in one study exposure to natural disasters was associated with increased connectedness (Walters et al., 2014) and in the other the opposite was true (Brown, 2017)—the research questions themselves are emblematic of an interest in the negative side of contact with nature. Notably, this interest in the potential negative side of contact with nature in terms of its effect on connectedness is similar to research on biophobia (Zhang et al., 2014a); however, research specifically focusing on the extent to which contact with aversive elements of nature promotes or undermines including nature in our sense of self or feeling a sense of oneness with nature is still needed.

Both a framework for explaining null effects and predictions about when contact with nature might have a negative effect are important for the development of theories describing the formation of connectedness. Future research can provide answers to these types of questions, and, subsequently, a better theoretical understanding of the different forms of contact with nature. However, it will require a shift from primarily looking for *whether* contact with nature promotes connectedness to explicitly examining instances in which contact with nature either does not promote connectedness or actively diminishes it as well as a focus on developing theories to explain null and/or negative effects.

Additionally, this section highlights that there are unanswered questions concerning comparisons between types of contact with nature. The presence of research showing that features of the environment matter when it comes to connectedness (e.g., Duffy and Verges, 2010; Wyles et al., 2019) indicates that contact with nature is likely a heterogenous category which may contribute to variability in the degree to which contact with different types of nature will affect connectedness (c.f., Duffy and Verges, 2010). If fostering connectedness is a goal, it is important to understand what features of the environment influence the degree to which contact with nature influences connectedness. Though the effects of actual first-hand contact with nature have received considerable attention more research is needed to know whether certain types of contact with actual nature have differential effects on connectedness—for example, blue spaces vs. rural green spaces or urban green spaces (c.f., Wyles et al., 2019). We also see few studies comparing videos of nature to actual nature (c.f., Mayer et al., 2009, S2–3) and none comparing VR and actual nature.

Last, despite research on contact with nature focusing on both adults and children, we do not know during which period of life contact with nature is *most* important. Although current evidence, noted above, suggests that adult contact is more important than childhood contact (e.g., Rosa et al., 2018), asking which type of contact—child or adult—begs the question of whether the distinction is meaningful.

In sum, research investigating the effects of contact with nature on connectedness should take the next steps and begin to explore (1) the heterogeneity of contact with nature (2) across the lifespan as well as begin focusing on (3) when—and subsequently why—contact with nature fails to promote, or actively suppresses, connectedness.

#### Activities

A handful of activities promote connectedness. These include activities where people are in direct contact with nature such as outdoor pastimes done for pleasure (e.g., Beery, 2013). Others include those which can potentially occur with only indirect exposure to nature, such as environmental educational programs (e.g., Liefländer et al., 2013). Yet others—including meditation (e.g., Aspy and Proeve, 2017) and the use of psychedelics (e.g., Nour et al., 2017)—can occur without any contact with nature at all.

##### Activities as More Than Contact With Nature

Activities that involve some degree of contact with nature—such as gardening (e.g., Beery, 2013; Sanguinetti, 2014), planting trees (e.g., Whitburn et al., 2019), walking dogs (Beery, 2013; Wyles et al., 2019), having picnics in nature, studying plants and animals (Beery, 2013) depicting nature artistically (Bruni et al., 2017), or receiving interpretation while touring nature parks (Burbach et al., 2012)—are positively associated with connectedness. For many of these activities it is unclear if the relations exist merely because these activities bring the individual into contact with nature. The alternative possibility is that the activities in some way enhance, or work independent of, the incidental contact with nature. Unfortunately, the extant literature does little to help dissociate these possibilities. One study, however, suggests that noticing nature increases connectedness and this effect seems to be above and beyond contact with nature; even though all participants spent equal time in nature, compared to business as usual, participants instructed to notice nature experienced increased connectedness (Passmore and Holder, 2017). This study, as an example, highlights the possibility that activities that *involve* contact with nature might be more than *just* contact with nature.

Just as not all contact with nature enhances connectedness, not all activities that involve contact with nature increase connectedness. Indeed, some activities, such as going to the beach and playing on a playground were not correlated with connectedness (Bruni and Schultz, 2010, S3) and other activities such as waterskiing and wake boarding (Beery, 2013), as well as exercising or playing in nature (Wyles et al., 2019) have been reported to be negatively correlated with connectedness. One possibility is that these activities involve treating nature as merely an arena in which to engage in the activity which could potentially result in nature becoming a non-salient background element of the experience or may even result in viewing nature from an entirely different perspective, where nature is objectified as a means to an end. Regardless, that these activities—which involve contact with nature—still seem to decrease connectedness suggests that activities can have an effect that is independent of the effect of contact with nature.

Environmental education is one commonly studied activity appearing in the connectedness literature. The majority of evidence appearing in this review suggests that participation in environmental education programs—which does not necessarily entail direct physical contact with nature (e.g., Lankenau, 2018)—is associated with increases in connectedness (Mayer and Frantz, 2004; Clayton et al., 2011; Liefländer et al., 2013; Sellmann and Bogner, 2013; Johnson-Pynn et al., 2014; Braun and Dierkes, 2017; Crawford et al., 2017; Otto and Pensini, 2017; Cho and Lee, 2018; Lankenau, 2018; Dopko et al., 2019). However, as with contact with nature, there are exceptions, with some studies showing no effect of participation in environmental education (e.g., Ernst and Theimer, 2011). And, again, as with contact with nature, the reasons for these disparities are not well outlined in the literature. We do not know what kinds of programs—classroom vs. field, broad verses specific, etc.—are most likely to increase connectedness. There is some evidence, however, that longer programs are more effective at fostering connectedness (e.g., Johnson-Pynn et al., 2014; Braun and Dierkes, 2017). This effect may be attributable to several things such as more impactful content, more immersion, or some other element that differs between longer and shorter programs, but the exact reason for this effect requires further research.

##### Activities Without Contact With Nature

There are also other activities which can promote connectedness that have both the potential to influence how we think about nature and do not necessarily involve actual contact with nature.

###### Meditation

Meditation, which alters how we think (c.f., Lutz et al., 2007), is one activity that can increase connectedness without actually being in nature. Formally, the western understanding of meditation is that it is a set of practices generally designed to cultivate particular mental qualities through repeated induction of a mental state (Lutz et al., 2007). A commonly known form of meditation is mindfulness meditation; however, there are other kinds of meditation and meditative practices that do not focus on mindfulness.

Indeed, meditation (Beery, 2013; Unsworth et al., 2016; Nisbet et al., 2019) and yoga (Beery, 2013) may enhance the effects of spending time in nature on connectedness; individuals who spent time meditating in nature felt greater connectedness than individuals who just spent time in nature (Unsworth et al., 2016; Nisbet et al., 2019). The effect of meditation, however, might not require contact with nature. For example, compared to progressive muscle relaxation mindfulness meditation and loving kindness meditation have been associated with connectedness without any contact with nature at all (Aspy and Proeve, 2017). This suggests that meditative practices likely have effects entirely separate from contact with nature. Similar to meditation, Langerian (see Langer, 2000) mindful learning—which is explicitly aimed at fostering a more flexible and open mindset (Tang et al., 2017) as well as at shifting thinking patterns (Wang et al., 2016)—has been associated with higher levels of connectedness compared to other forms of learning (Wang et al., 2016, 2019).

###### Reflection

In the abstract, meditation has a great deal to do with reflective modes of thinking, and, as stated at the outset of this paragraph, focuses on altering how we think. Meditation, however, is not the only way of altering the way we think nor is it the only way of engaging in reflection. Importantly, there is evidence that the way in which we reflect upon past experiences (e.g., eudaimonic vs. hedonic reflection vs. mundane recollection) may influence our sense of connectedness (Lengieza et al., 2021). This suggests that other forms of reflective or contemplative practices beyond meditation may also impact our feelings of connectedness. Additionally, there are more explicit ways to alter how we think in the moment; in some cases, we can consciously choose to think about nature in a different light. For example, anthropomorphizing nature is associated with increased connectedness, an effect corroborated experimentally (Tam et al., 2013; Liu et al., 2019). Thus, there is a growing body of evidence that altering the way we think (e.g., meditation, mindful learning) and what we think about (e.g., the content of reflections, anthropomorphized nature) has the potential to increase connectedness.

###### Psychedelics

Last, recent research suggests that lifetime use of psychedelics—known for their capacity to alter ways of thinking about the world (Pollan, 2018)—is associated with increased connectedness (Forstmann and Sagioglou, 2017; Nour et al., 2017). Further, a preliminary experimental study—with an admittedly small sample—demonstrated that connectedness increased following a guided psilocybin therapy session (Lyons and Carhart-Harris, 2018). Yet, other common recreational drugs have either not shown an association with connectedness (e.g., Forstmann and Sagioglou, 2017), or have been negatively associated with connectedness (e.g., cocaine and alcohol use, Nour et al., 2017), suggesting that the positive effect is possibly specific to psychedelics.

##### Summary and Critique of Research on Activities

There is evidence that a variety of activities enhance connectedness. This includes activities such as gardening or planting trees (e.g., Whitburn et al., 2019), participating in environmental education (e.g., Lankenau, 2018), as well as meditating (e.g., Aspy and Proeve, 2017) and certain recreational drug use (Forstmann and Sagioglou, 2017).

In addition to replicating findings and experimentally confirming correlational relationships, future research should address the question, already noted in this section, of whether the activities that involve contact with nature reviewed in this section have an effect above and beyond the effect of having contact with nature and if so, why. While some of these activities can involve varying degrees of contact with nature, it seems unlikely that they can be boiled down to simply be about being in nature. Instead, it is likely that they enhance the contact with nature that happens to be involved in that activity. For example, gardening (c.f., Beery, 2013) also includes a component of taking care of or nurturing nature. Does this additional facet of caring for nature bring something new to the table or can it really be reduced to being in contact with nature? The need to understand whether nature-based activities have affects above and beyond contact with nature is also particularly apparent for activities that seem to suppress connectedness despite involving contact with nature such as exercising or playing in nature (Wyles et al., 2019) or waterskiing and wake boarding (Beery, 2013). Research is needed to understand why these activities overshadow the effects of contact with nature.

Finally, there may be moderators of the relationship between various activities or experiences and connectedness. For example, age may moderate the effect of regular participation in outdoor activities and connectedness; regular participation—vs. non-regular participation—may only matter for older age groups (Beery, 2013). As illustrated in the next section, age is only one of many individual differences that may be worthy of consideration. Few studies, though, have investigated the possibility of factors that moderate the relationship between connectedness and the experiences and activities listed above. Therefore, research on the effect of various activities on connectedness should pay attention to potential moderators.

### Individual Differences That Influence Connectedness

Several individual differences are associated with connectedness. Some of these individual differences, such as age, gender, race, and socioeconomic status, are generally considered demographics. Others fall neatly into the category of personality. The remaining individual differences considered here are best characterized as various types of worldviews. While less practically manipulable compared to other antecedents identified in this review, it is still valuable to consider the associations between individual differences and connectedness because they illuminate for whom certain experiences might differentially affect connectedness and they may also stimulate discussions about which psychological processes might be informative to study. More specifically, these individual differences may provide insight into the influence of social processes on connectedness, such as self-selection into contact with nature or socialization which encourages developing a connection to nature. Further, although individual differences do not lend themselves to direct intervention, it is probable that some of them moderate the influence of other antecedents of connectedness, making them of practical interest.

#### Demographics

Demographics, as antecedents to connectedness, are important to consider because they can provide boundary conditions for findings and can potentially provide insights into self-selection into experiences that would influence contact with nature, for example.

##### Age

Research on age suggests that our sense of connectedness might be influenced by where we are in our lifespan. Several studies with adults indicate that age is positively associated with connectedness (Burbach et al., 2012; Beery, 2013; Sanguinetti, 2014; Zhang et al., 2014b, S1–2; Harvey et al., 2016; Lumber et al., 2017; Nour et al., 2017; Diessner et al., 2018). However, a sizeable number of studies with adults suggest no relationship (Mayer and Frantz, 2004; Dutcher et al., 2007; Bruni et al., 2008; Weinstein et al., 2009, S1–3; Walters et al., 2014; Unsworth et al., 2016, S1–2; Swami et al., 2016; Brown, 2017; Whitburn et al., 2019). In contrast, studies examining school-aged children indicate that age has the opposite effect amongst children; younger children tend to feel greater connectedness than older children (Liefländer et al., 2013; Braun and Dierkes, 2017; Crawford et al., 2017; Larson et al., 2018). These opposing patterns suggest a curvilinear effect whereby children temporarily grow-out of their connection to nature, so to speak, until at some point they begin to reformulate their connection to nature. At present, this pattern has been supported by one study specifically seeking to sample a range of ages to address the question of age’s impact on connectedness more comprehensively (Hughes et al., 2019).

##### Gender

When gender differences in connectedness are found, they appear to more often conclude that women feel greater connectedness than men (Schultz and Tabanico, 2007, S3–4; Mayer et al., 2009, S2; Bruni and Schultz, 2010, S3; Beery, 2013; Sanguinetti, 2014; Zhang et al., 2014b, S1; Pensini et al., 2016; Spendrup et al., 2016; Swami et al., 2016; Crawford et al., 2017; Nour et al., 2017; Hughes et al., 2019) than they report that men feel more connectedness than women (Larson et al., 2018; Wyles et al., 2019). However, a number of studies that provide information about the associations between gender and connectedness report no effect (Mayer and Frantz, 2004, S1–2; Frantz et al., 2005; Bruni et al., 2008; Mayer et al., 2009, S1 and S3; Weinstein et al., 2009, S1–3; Bruni and Schultz, 2010, S1–2; Duffy and Verges, 2010; Vess et al., 2012; Zhang et al., 2014b, S2; Barton et al., 2016; Davis and Stroink, 2016a,b; Harvey et al., 2016; Unsworth et al., 2016, S1–2; Lumber et al., 2017; Di Fabio and Kenny, 2018; Diessner et al., 2018; Liu et al., 2019; Whitburn et al., 2019). Additionally, there is no evidence that gender moderates any effects in any of the studies reporting on gender and connectedness (e.g., Mayer et al., 2009; Duffy and Verges, 2010; Vess et al., 2012; Capaldi et al., 2014). Thus, the conclusion to be drawn from the literature is that, if gender has an effect at all—a tenuous association at best—women may feel greater connectedness than men.

##### Other

There are three understudied demographics—education, race, and socioeconomic status—which may be worth further attention. Several studies have found that level of education does not have an effect on connectedness (Mayer and Frantz, 2004, S1; Dutcher et al., 2007; Beery, 2013; Walters et al., 2014; Nour et al., 2017; Whitburn et al., 2019); however, other studies have found higher education to be associated with lower connectedness (Sanguinetti, 2014; Brown, 2017). Similarly, a small number of studies have found no relationship between race and connectedness (Weinstein et al., 2009, S1–3; Whitburn et al., 2019), while one found that white participants report a sense of connectedness more so than non-white participants (Larson et al., 2018). And, again similarly, while studies have suggested no relationship between connectedness and socioeconomic status (Wyles et al., 2019) or income (Mayer and Frantz, 2004; Dutcher et al., 2007; Beery, 2013; Walters et al., 2014), yearly income and home ownership have been associated with decreased connectedness (Whitburn et al., 2019). Thus, based solely on the literature appearing in this review, it would be premature to draw conclusions about the effect of level of education, race, and socioeconomic status on connectedness.

#### Personality

Certain facets of personality appear to exert an influence on connectedness. The most frequently reported relation is a positive association between openness to experience and connectedness (Nisbet et al., 2009; Tam, 2013; Brick and Lewis, 2014; Zhang et al., 2014b, S1; Lee et al., 2015; Richardson and Sheffield, 2015; Di Fabio and Bucci, 2016; Forstmann and Sagioglou, 2017; Nour et al., 2017). Additionally, openness is the only personality facet shown to correlate with connectedness when using observer reports of personality (Lee et al., 2015). There is also strong evidence that agreeableness (Nisbet et al., 2009; Tam, 2013; Brick and Lewis, 2014; Zhang et al., 2014b, S1; Di Fabio and Bucci, 2016) and conscientiousness (Nisbet et al., 2009; Tam, 2013; Brick and Lewis, 2014; Zhang et al., 2014b, S1; Di Fabio and Bucci, 2016; Forstmann and Sagioglou, 2017) are positively associated with connectedness. While the other facets of personality—specifically, humility (Brick and Lewis, 2014; Lee et al., 2015), emotionality (Tam, 2013; Brick and Lewis, 2014), extraversion (Nisbet et al., 2009, S1; Tam, 2013; Zhang et al., 2014b, S1) and (less) neuroticism (Zhang et al., 2014b, S1)—have shown positive correlations with connectedness, the evidence is not as overwhelming (i.e., only one or two studies showing significant associations per personality attribute).

#### Worldviews

Worldviews—which encompass beliefs, attitudes, orientations, and values (Clayton and Myers, 2015)—are associated with connectedness. While demographics and personality are determined at an early age and are largely immutable, worldviews develop over time and consequently have a greater degree of mutability. Therefore, worldviews may be additional targets for efforts to enhance connectedness. Research in this domain is important because, as noted in the preceding sections, it can help us identify potential moderators of other antecedents of connectedness. This type of research may also inform theoretical accounts of connectedness. For example, if connectedness consistently covaries with a particular class of constructs, such as those associated with self-transcendence, then we can more confidently conceptualize connectedness as a form of self-transcendence and make predictions based on that view.

##### Common Worldviews and Connectedness

As might be expected, people who hold more positive environmental beliefs feel greater connectedness (Mayer and Frantz, 2004, S1–2; Frantz et al., 2005; Nisbet et al., 2009, S1; Bruni and Schultz, 2010; Clayton et al., 2011, S1; Davis et al., 2011; Brick and Lewis, 2014; Lee et al., 2015; Davis and Stroink, 2016a,b; Whitburn et al., 2019). Additionally, higher levels of connectedness are found among individuals who tend to appreciate natural beauty (Zhang et al., 2014b, S1–2; Capaldi et al., 2017, S1–2; Lumber et al., 2017; Diessner et al., 2018), are more politically liberal (Dutcher et al., 2007; Nour et al., 2017), and are more empathic (Mayer and Frantz, 2004, S2 and S4; Di Fabio and Bucci, 2016; Di Fabio and Kenny, 2018). Lower levels of connectedness are found among those who are more politically conservative (Brick and Lewis, 2014) more authoritarian (Nour et al., 2017), more oriented toward consumerism (Mayer and Frantz, 2004, S4) or materialism (Hedlund-de Witt et al., 2014), and who ascribe to the feminine beauty ideal (Scott, 2010, S1–2). In general, individual’s values seem to be associated with connectedness (Sellmann and Bogner, 2013; Lumber et al., 2017), an effect which may be initially derived from parent’s values (Cheng and Monroe, 2012). There is also evidence that religious fundamentalism is negatively associated with connectedness; however, this was only under conditions of mortality salience (Vess et al., 2012), whereas more general religiosity or spirituality may have either no (Vess et al., 2012, S1–3, but see Brown, 2017) or a positive effect on connectedness (Hedlund-de Witt et al., 2014).

##### Self-Transcendent Worldviews and Connectedness

Connectedness also shows positive associations with constructs that support the perspective that connectedness reflects a form of self-transcendence. Specifically, connectedness is positively associated with self-transcendent values and negatively associated with self-enhancement values (Tam, 2013). Further, connectedness is positively associated with constructs involving connecting to something greater, such as connectedness to community (Sanguinetti, 2014) and even to humanity as a whole (Lee et al., 2015; Lengieza et al., 2021), as well as with greater moral expansiveness (Crimston et al., 2016) and more altruism (Nisbet et al., 2009, S1). Moreover, connectedness is positively associated with non-self-interested concern for nature (e.g., biospheric concern; Mayer and Frantz, 2004, S4–5; Davis and Stroink, 2016a,b; although this effect is sometimes not found, e.g., Schultz and Tabanico, 2007, S2; Duffy and Verges, 2010) whereas, at best, connectedness is simply not associated with self-interested concern for the environment (e.g., egoistic concern; Mayer and Frantz, 2004, S4; Schultz and Tabanico, 2007, S1–2; Duffy and Verges, 2010; Davis and Stroink, 2016a,b) and, at worst, may be negatively associated with such self-centered concern (Mayer and Frantz, 2004, S5; Schultz and Tabanico, 2007, S1). Lastly, individuals who think more in terms of systems—which is related to seeing oneself as part of a set of interrelated parts and suggestive of self-transcendence (c.f., Lengieza et al., 2021)—tend to report higher levels of connectedness (Davis and Stroink, 2016a). Thus, there is evidence that constructs consistent with self-transcendence (e.g., self-transcendence values; Tam, 2013) are associated with connectedness which supports the contention that connectedness reflects a form of self-transcendence (e.g., Lengieza et al., 2021).

#### Summary and Critique of Research on Individual Differences

Who we are and how we view the world influences our sense of connectedness. The apparent consensus is that age is associated with connectedness; however, this effect is likely curvilinear (see Hughes et al., 2019). Gender on the other hand, may or may not be related to connectedness, at least directly, as many studies reported no gender related effects. Still, a non-negligible number of studies have reported that women feel greater connectedness than men, suggesting that there may be an effect of gender, though one that is likely small. In both cases, however, a more systematic investigation into the effects of age and gender seems warranted at this juncture. Individual’s personality also influences connectedness, most notably their degree of openness to experience (e.g., Lee et al., 2015) as well as agreeableness and conscientiousness are associated with connectedness (e.g., Brick and Lewis, 2014). Lastly, there are a multitude of worldviews which are associated with connectedness (e.g., Crimston et al., 2016), several of which are consistent with viewing connectedness as a form of self-transcendence (e.g., Lengieza et al., 2021).

It is not necessarily surprising that certain types of individuals, those from certain backgrounds, and those who hold certain views more (or less) easily develop a connection to the natural world than others. For the most part, however, it is unclear exactly *why* various demographic characteristics and facets of personality would influence connectedness, largely because of a lack of theory. There are several potential reasons that demographics might be theoretically important. Demographics memberships might serve as proxies for likelihood of having contact with nature, for example. That is, certain individuals may be more or less likely to have contact with nature, and therefore, end up feeling lower connectedness. Alternatively, potential differences between demographic groups could be a matter of socialization and what is culturally valued by one’s peers. Perhaps in some socio-demographic contexts individuals are encouraged to connect with nature—or at least encouraged to pay attention to their relationship with nature—whereas in others, individuals do not receive such encouragement. To empirically test these possibilities, however, researchers will need to begin considering the mediating process through which these variables influence connectedness. To date, one study suggests that part of the reason agreeableness and openness might be associated with connectedness is *via* empathy (Di Fabio and Kenny, 2018). However, empathy is unlikely to be the only pathway between personality and connectedness and, thus, more research is undoubtedly warranted. Further investigation into why these relationships exist—to the extent that they do—is theoretically helpful because it may shed light on potential social processes that influence connectedness and may also inspire investigations into other individual differences that might influence connectedness.

Additionally, individual differences are likely candidates for moderators of other antecedents of connectedness. For example, as noted in a previous section, age potentially influences the importance of participation in outdoor activities with activities being more important for older adults than younger adults (Beery, 2013) and, additionally, one meta-analysis suggests the effect of mindfulness on connectedness is larger in samples with older participants (Schutte and Malouff, 2018). More generally, certain individuals may tend to experience the same activity differently. Practically, these considerations could influence how one might construct interventions to increase connectedness. For example, individual differences in appreciating natural beauty might influence the effect of engaging with nature artistically (c.f., Bruni et al., 2017). Thus, if one were to design an intervention meant to enhance connectedness through artistic engagement with nature, it might make sense to first focus on fostering an appreciation of natural beauty prior to the core focus of the intervention.

### Psychological States That Influence Connectedness

Contrasting with relatively stable individual differences noted above, some studies identify more malleable and transitory underlying psychological states that may promote connectedness. Ultimately, research on these states provides insight into the psychological processes through which other antecedents of connectedness may have their effect. To the extent that we understand the process that unfolds behind a given antecedent, such as contact with nature, we can better activate that process to make the experience, or other antecedent, as impactful as possible. Thus, research on psychological states that promote connectedness will inform what to leverage in efforts to increase connectedness. Additionally, as was noted in the worldviews section, this research may help form theoretical accounts of connectedness. Similar to worldviews, if certain classes of psychological states, such as states involving the self, tend to consistently covary with connectedness then we can more confidently conceptualize connectedness as a phenomenon involving the self. The states reviewed in this section can be categorized as being related to mindfulness, the self, and affect.

#### Mindfulness

While meditation, as an activity, was mentioned earlier in this review, not all meditative practices are aimed at increasing mindfulness (e.g., loving-kindness meditation). Therefore, conflating meditation and mindfulness should be avoided. Additionally, there is a great deal that meditation might change even when it is aimed at increasing mindfulness. Thus, simply because meditation influences connectedness does not necessarily mean that mindfulness, as psychological quality of mind, influences connectedness. For example, meditation may simply increase individuals’ ability to introspect—which is not the same as mindfulness—and this increase in introspection might be the true cause of some hypothetical increase in connectedness (c.f., Richardson and Sheffield, 2015). Research solely examining the practice of meditation also cannot illuminate which facets of mindfulness are associated with connectedness. It is, therefore, necessary to also measure mindfulness to determine whether, and how, mindfulness as a psychological quality impacts connectedness.

Several studies demonstrate a positive link between mindfulness and connectedness (see Schutte and Malouff, 2018, for a meta-analysis). Given the meta-analysis on the subject, it seems unnecessarily redundant to outline all of the individual findings related to the mindfulness–connectedness association; however, it is worth noting one important trend. While mindfulness in general has been associated with higher levels of connectedness (e.g., Howell et al., 2011; Richardson and Sheffield, 2015, S1–2; Unsworth et al., 2016, S1; Schutte and Malouff, 2018), certain facets seem to be more related to connectedness than others. Specifically, the “observing” (Barbaro and Pickett, 2016, S1–2; Hanley et al., 2017), “nonreactivity” (Barbaro and Pickett, 2016, S1–2; Hanley et al., 2017), and “describing” (Barbaro and Pickett, 2016, S1–2) facets of mindfulness have been associated with connectedness, whereas the “nonjudging” (Barbaro and Pickett, 2016, S1–2; Hanley et al., 2017) and “acting” (Barbaro and Pickett, 2016, S1; Hanley et al., 2017) facets have not.

##### Summary and Critique of Research on Mindfulness

The obvious consensus in the literature—largely informed by the results of the meta-analysis (i.e., Schutte and Malouff, 2018)—is that the psychological experience of mindfulness is associated with increased connectedness. This association in conjunction with the evidence that meditation also increases connectedness, seems to suggest that changes in mindfulness would mediate the relationship between meditation and connectedness. To date, however, this process of mediation has not been empirically tested. Thus, future research should attempt to document the process through which meditation increases connectedness. Such an endeavor will entail paying greater attention to various facets of mindfulness to fully understand the relations between meditation, mindfulness, and connectedness.

It is also possible that mindfulness is not the only path through which meditation increases connectedness; perhaps meditation also influences connectedness through an effect on the way we think about the self (c.f., Hanley et al., 2017) through an effect on affective experiences (c.f., Jazaieri et al., 2013), or simply through increased reflective or introspective propensity (c.f., Richardson and Sheffield, 2015; see ensuing sections for elaboration). Consequently, research investigating the process through which meditation has its effect should consider alternative mechanisms in addition to mindfulness.

#### Psychological States Related to the Self

Connectedness is defined as including nature in the self, and, therefore, it is reasonable to expect that psychological states associated with the self would influence connectedness (Lengieza and Swim, 2021). Thus, to support the assertion that connectedness does involve including nature in the self, it is important to study if, and how, self-related psychological phenomena impact connectedness.

##### Negative Impacts of the Self on Connectedness

Self-awareness, with its different facets, is one such self-related phenomena that affects connectedness. Studies suggest that taking oneself as the object of awareness might negatively impact connectedness. In one study, self-objectification—taking the critical perspective of an observer when considering the self—was negatively associated with connectedness across three samples of women (Scott, 2010). Another study demonstrated that being seated in front of a mirror—which theoretically increases objective self-awareness—diminished connectedness (Frantz et al., 2005). This suggests that objective self-awareness may interfere with connectedness.^4^

Other studies corroborate the findings from research on objective self-awareness. Evidence suggests that being publicly self-aware—being more aware of how you appear to others—is negatively associated with connectedness (Mayer et al., 2009). In line with the negative association with public self-awareness, rumination—which was defined as anxious, or preoccupied, attention that is focused on the self and is concerned with self-worth or failure (making it similar to public self-awareness)—was negatively correlated with connectedness (Richardson and Sheffield, 2015). Further, research suggests that decreases in public self-awareness may be the mechanism through which contact with nature increases connectedness (Lengieza and Swim, 2021). Thus, being overly focused on oneself from a third-person perspective seems to have a negative impact on connectedness.

In addition to research on self-awareness, other evidence implies that diminishing overly self-focused attention may be important for facilitating connectedness. First, some studies investigating mindfulness implicate the self as an important determinant of connectedness; one of the reasons that mindfulness may influence connectedness is because of its effect on decentering (Hanley et al., 2017; see also Nisbet et al., 2019). Specifically, decentering has been argued to be linked with self-transcendence and to a blurring of the self–other dichotomy (Hanley et al., 2018). Thus, the link between decentering and connectedness is consistent with the defining connectedness as a form of self-transcendence (Lengieza et al., 2021). Second, one of the reasons that psilocybin increases connectedness may be because of the effect it has on individual’s sense of self; ego dissolution—a pharmacologically induced state of selflessness associated with psychedelics—during individuals’ most significant experience with psilocybin was associated with increased connectedness (Nour et al., 2017). Thus, this evidence would loosely suggest that diminishing attention to the self might promote connectedness.

##### Positive Influences of the Self on Connectedness

There is other evidence that which implicate the self in the formation of connectedness and also suggests that the self might not always be an obstacle in the way of forming a connection with nature. In contrast to objective self-awareness and public self-awareness, private self-awareness—being aware of one’s inner experience, effectively synonymous with introspection—may enhance connectedness (Mayer et al., 2009). Consistent with this finding, reflective self-attention was found to be a better predictor of connectedness than mindful attention (Richardson and Sheffield, 2015, S1–2) and moderated the effect of exposure to nature, with higher levels of reflective self-attention strengthening the effects of contact with nature on connectedness (Richardson and Sheffield, 2015, S3). Additionally, as noted above, one of the mechanisms through which VR might increase connectedness may be body transference (Ahn et al., 2016, S1–3); in other words, a transfer of our corporeal sense of self seems to influence connectedness. Fourth, the ways in which we construe the self (e.g., interdependent, independent, etc.) seem to influence connectedness (Davis and Stroink, 2016b) as does the way that we feel about ourselves (i.e., self-esteem; Zhang et al., 2014b, S2; Swami et al., 2016). Thus, there is growing evidence that self-related phenomena are, at the very least, an important part of the formation of connectedness.

##### Summary and Critique of Research on Psychological States Related to the Self

The above evidence concludes that self-related phenomena play a critical role in the formation of connectedness. Specifically, both the way in which we attend to the self (e.g., Richardson and Sheffield, 2015) and the way we subjectively experience the self (e.g., Hanley et al., 2017; Nour et al., 2017) influence connectedness, though there is still much of this story left to untangle.

This means, however, that there are many exciting directions for future research in this area. First, there are several additional phenomena related to the self that may impact individuals’ sense of connectedness such as goal-related content (e.g., actual, ideal and ought selves; Higgins, 1987) temporal reflections (e.g., past, present and future selves; Markus and Nurius, 1986) and structure of the self (e.g., self-schemas, Markus, 1977). The growing evidence that the self influences connectedness in multiple ways suggests that the unstudied associations between these facets of the self and connectedness warrants future investigation.

Second, there are opportunities to further differentiate already-studied phenomena (c.f., public vs. private self-awareness) to create more nuanced accounts of how self-related psychological states are expected to impact connectedness. As an example, the observation of a positive association between connectedness and private self-awareness in contrast to the negative association with public self-awareness suggests that these two types of self-awareness should be differentiated. Moreover, these specific opposing relations raise two interrelated points. (A) These opposing effects emphasize that more research is necessary to understand when focusing on the self diminishes connectedness and when it might benefit it. (B) It seems to tentatively suggest that what gets in the way of connecting to nature is a disproportionate (e.g., Lengieza and Swim, 2021) and preoccupied (e.g., Richardson and Sheffield, 2015) focus on the self—as opposed to an introspective or proportionate focus on the self (e.g., Richardson and Sheffield, 2015). In other words, in the context of its relation to connectedness, there may be such a thing as a healthy and unhealthy focus on the self.

Third, there is the opportunity to use research on the self to inform research on the antecedents of connectedness. For example, it is worth considering how self-related psychological states might mediate relations described in earlier sections. One could test, as suggested above, whether decentering—which tentatively links mindfulness to connectedness (e.g., Hanley et al., 2017)—is one of the avenues through which meditation affects connectedness. Further, it is also worth considering how other, yet-studied, situations or interventions that are believed to influence the self might be related to changes in connectedness. For example, body scan meditation has been shown to induce a blurring of the self-other boundary (Dambrun, 2016), thus studying the effect on connectedness that this type of intervention has might warranted solely on the basis that it influences the self.

#### Affect and Motivation

Affective states have an impact on individuals’ sense of connectedness. A meta-analysis suggests that, on the whole, positive affect as well as wellbeing are positively correlated with connectedness (Capaldi et al., 2014) and research has consistently concluded that positive affect promotes connectedness (e.g., Mayer et al., 2009, S1–3; Howell et al., 2011, S2; Nisbet et al., 2011, 2019, S1–3; Nisbet and Zelenski, 2011, S1–2; Capaldi et al., 2014, 2017, S1; Dopko et al., 2014, 2019, S1; Crawford et al., 2017). In fact, increased positive affect may be one of the psychological mechanisms through which contact with nature increases connectedness (Nisbet et al., 2011), although not all studies find significant relationships between affect and connectedness (e.g., Schultz and Tabanico, 2007, S5; Howell et al., 2011, S1; Vess et al., 2012).

Research also suggest that it is useful to delineate different types of positive affect; specific forms of positive affect, such as awe (Yang et al., 2018; Nisbet et al., 2019) or similar types of emotions such as elevating experiences (Capaldi et al., 2017, S1; Lengieza et al., 2021) have been positively associated with connectedness. Moreover, meaning and purpose, a form of eudemonic affect, is positively correlated with connectedness (Hinds and Sparks, 2009; Howell et al., 2011, S1–2; Nisbet et al., 2011, S1 and S3; Capaldi et al., 2017, S1) as are other forms of more general wellbeing (Capaldi et al., 2014, 2017, S1–2; Richardson et al., 2016; Nisbet et al., 2019) whereas hedonic affect was no longer associated with connectedness after controlling for eudaimonic affect (Lengieza et al., 2021).

In addition to focusing on positive affect, a few studies have also shown that negative affect is negatively correlated with connectedness (Mayer et al., 2009, S2; Nisbet et al., 2011; Nisbet and Zelenski, 2011, S4; Dopko et al., 2019). As noted in the above sections, there are open questions about whether “the bugs and mud” of nature negatively impacts connectedness. To the extent that “bugs and mud” elicits negative affect, it would be reasonable to expect them to negatively impact connectedness.

Motivational factors may also influence connectedness. One study found that after experiencing or recalling ostracism individuals reported a stronger disposition to connect with nature (Poon et al., 2015, S1 and S3), suggesting our more universal needs for relatedness (Ryan and Deci, 2000) might be a potential determinant of connectedness. Another study, referenced above, found connectedness was lower among individuals for whom thoughts about death were more accessible, suggesting that our purported motivation to avoid our own mortality may hinder connectedness (Vess et al., 2012).

##### Summary and Critique of Research on Affect

Affect may be an important determinant of connectedness. Positive affect has been shown to (e.g., Nisbet and Zelenski, 2011) promote connectedness, whereas negative affect appears to suppress it (e.g., Nisbet et al., 2011). For the most part, however, few studies consider affect and emotions with increased granularity. That is, most studies treat positive affective states and negative affective states as cohesive groups. Affective states, however, differ on more than just valence (e.g., approach–avoidance; Harmon-Jones et al., 2017) and it may be worthwhile to understand how certain classes of emotions affect connectedness (e.g., awe as a type of self-transcendent emotions; Stellar et al., 2017) as there is evidence that different types of positive affect differentially predict connectedness (Lengieza et al., 2021).

## Discussion

The purpose of this review was to provide an overview of the literature on the psychological antecedents of connectedness. At present, many studies point to the importance of actual and mediated contact with nature as well as to the importance of the activities that one does as antecedents of connectedness. People also seem to vary in connectedness based on individual differences which suggests that different life experiences or ways of engaging with the world may be influential factors that affect connectedness. Prime examples of these individual differences are age, openness to experience, as well as worldviews that reflect attitudes toward nature and self-transcendence. Other research has illuminated potential psychological processes that may explain other effects outlined in the review. For example, decreased public self-awareness (Lengieza and Swim, 2021) and increased positive affect (Nisbet and Zelenski, 2011) may mediate the effect of contact with nature on connectedness.

### Broader Critiques of The Literature

This review, however, highlights broader critiques of the literature, in addition to those raised in the summary sections throughout the paper. First, there appears to be a lack of theoretical frameworks detailing how connectedness forms. Development of such theories will help guide the generation of novel research questions and will also help guide the selection of potential moderators worthy of investigation. Theories are also important because they will help outline when effects are and are not expected to occur. Thus, such theories will consequently help provide frameworks to understand and interpret null findings—a point which is especially important for research on contact with nature. Additionally, theories are important to help unify seemingly disparate findings, such as the loose collection of work outlined in the worldviews section.

Second, the ability to develop such theories would greatly benefit from greater inquiries into the process through which known antecedents have their effects. While there are several antecedents believed to impact connectedness, we often do not know *why* these antecedents have their effect. For example, despite many studies testing whether contact with nature increases connectedness, only two studies have attempted to document the process through which this effect occurs (Nisbet and Zelenski, 2011; Lengieza and Swim, 2021). Understanding the process through which an effect occurs is not only scientifically interesting in its own right—being critically important for the development of theories—but also important for designing effective interventions for practical use. To illustrate the latter point, to the extent that the reason contact with nature has an effect on connectedness is, in part, because it decreases public self-awareness (Lengieza and Swim, 2021), then this would suggest an intervention in which individuals are taken to a natural area among a group of strangers might be less effective than individual excursions into nature because being surrounded by strangers would likely *increase* concerns about how one appears to others (i.e., public self-awareness).

### Specific Future Directions

There are several subfields within this body of research that are developed enough to warrant more nuanced investigations. The research on the mindfulness–connectedness association is a good example of why more nuanced accounts of a particular construct’s impact on connectedness is theoretically and practically valuable.

#### Nuances of the Mindfulness–Connectedness Relationship

There is substantial research that has demonstrated a link between mindfulness and connectedness, and we can be fairly confident that, generally speaking, mindfulness increases connectedness (Schutte and Malouff, 2018). A few researchers have taken the step toward a more nuanced account and investigated which of the many facets of mindfulness might be most responsible for this association (e.g., Barbaro and Pickett, 2016); the preliminary indication being that not all facets of mindfulness affect connectedness (e.g., Barbaro and Pickett, 2016). This is an important observation for both (a) theoretical accounts of the formation of connectedness and (b) for the design of effective interventions. Regarding the former, this is important because, if one can identify something common between the factors of mindfulness that are related to connectedness, it would be a step toward creating a parsimonious account of the influence of mindfulness on connectedness. Regarding the latter, there are a variety of interventions that one could employ to increase mindfulness, and they might not influence all facets of mindfulness in the same way. Thus, a given mindfulness intervention might not target one of the facets believed to impact connectedness. For example, sitting meditation seems to primarily increase the non-judging facet of mindfulness (Sauer-Zavala et al., 2013), which has not been correlated with connectedness (e.g., Barbaro and Pickett, 2016), whereas both body scan meditation and yoga seem to primarily increase the describing facet of mindfulness (Sauer-Zavala et al., 2013), which has been correlated with connectedness (e.g., Barbaro and Pickett, 2016). Therefore, an intervention using sitting meditation might not be the more effective means of promoting connectedness *via* mindfulness, at least compared to yoga or body scan meditation. Such an insight would be lost without this more nuanced view of mindfulness.

#### Other Areas in Need of Nuance

There are other sub-areas that would benefit from nuanced accounts of effect that their phenomena of interest have on connectedness: contact with nature, self-awareness, and affect.

##### Nuances of the Contact–Connectedness Relationship

While contact with nature seems to be an important determinant of connectedness only one study appeared in this review that compared different types of contact with nature within the same study (e.g., Wyles et al., 2019). There are a number of dimensions on which nature can vary—from manicured to wild, from green to blue to gray, from novel to familiar, etc.—and it would be valuable to know, for the same general reasons listed in the preceding mindfulness example, if and how these dimensions might matter.

##### Nuances of the Self-Awareness–Connectedness Relationship

Research on self-awareness would also benefit from increased consideration of the nuances between types of self-awareness. Specifically, it seems that public and private self-awareness have opposing effects on connectedness (e.g., Mayer et al., 2009), suggesting that there may be a type of focus on the self that is beneficial and a type of focus on the self that is not. However, at present, no research has truly addressed this question systematically.

##### Nuances of the Affect–Connectedness Relationship

Research on affect and connectedness may also benefit from differentiating between various forms of affect. As the literature currently stands, positive affect promotes, whereas negative affect diminishes, connectedness (e.g., Nisbet et al., 2011). There is reason to believe, however, that not all positive affect will affect connectedness in the same way; (e.g., eudaimonic vs. hedonic affect; Lengieza et al., 2021). Thus, it may be worthwhile to consider more nuanced distinctions between similar types of emotions.

#### Cultural Contexts

Finally, it may be appropriate, at this stage in the field, to begin considering these effects with an explicit cross-cultural lens. For example, the trend in connectedness across ages (Hughes et al., 2019) may not adhere to the same pattern for interdependent and independent cultures (Markus and Kitayama, 1991) to the extent that these age-related differences stem from differences in social pressures at different points in individuals’ lives. Additionally, as another example, thinking about or focusing on the self might not activate the same set of processes across cultures and therefore may impact connectedness differently (c.f., Zhu et al., 2007). Therefore, the relationship between connectedness and self-awareness (e.g., Lengieza and Swim, 2021) may differ between cultural contexts. Consequently, future research might want to consider if and how certain cultural contexts might affect connectedness directly, as well as might moderate the effect of other antecedents.

### Preliminary Theoretical Considerations

The sheer number of studies reporting the effect of contact with nature on connectedness may give the impression that contact with nature is *the* way to promote connectedness. However, there are several key findings that occur in contexts in the absence of contact with nature. Specifically, both loving-kindness and mindfulness meditation in the absence of contact with nature seem to promote connectedness (Aspy and Proeve, 2017). Additionally, lab-based manipulations of self-awareness affect connectedness in the absence of nature (Frantz et al., 2005). Further, mindful learning seems to increase connectedness *without* requiring contact with nature (e.g., Wang et al., 2019).

Upon closer inspection, these findings seem to support the tentative theoretical perspective that the default tendency is for people to develop a sense of connectedness. One can think of meditation, specifically mindfulness meditation, as being specifically aimed at minimizing problematic ways of thinking which ultimately create unnecessary pressures in our everyday lives (i.e., “clinging” and “aversion”). According to theories of self-awareness, public self-awareness should increase the influence of external standards whereas private self-awareness should increase the influence of internal standards (Govern and Marsch, 2001; Carver, 2012). Thus, the former seeming to inhibit connectedness and the latter seeming to promote it, suggests that—at least in in a psychological vacuum—the default tendency may be toward increasing connectedness. This would be consistent with the biophilia hypothesis (see Wilson, 1984; Kahn Jr, 1997) which proposes that we have an innate affinity other forms of life and for nature broadly. Further, the fact that connectedness appears to decrease heading into adolescence—a period of time during which self-construals appear to more heavily rely on external pressures (i.e., other’s impressions; e.g., Pfeifer et al., 2009), at least in the western context—and steadily increases afterward, is consistent with the view that connectedness thrives in the absence of counter self-preoccupied pressures. Thus, our tentative suggestion is that people innately develop connectedness and that other pressures—which are presumably common in modern-day life—may often work against that innate tendency. Indeed, it may be that contact with nature simply represents a return to that which feels normal, a brief reprieve from all the concerns and external pressures of everyday life that keep us disconnected from nature (c.f., Lengieza and Swim, 2021).

This view is also consistent with other perspectives found in psychology more broadly. Specifically, the need for relatedness (Ryan and Deci, 2000) or belonging (Baumeister and Leary, 1995) and the need for self-expansion (Aron and Aron, 1986; Aron et al., 2013) are both believed to be fundamental motives that drive human experience and behavior. Connectedness may fulfil both needs (e.g., Mayer and Frantz, 2004) which would be consistent with viewing greater connectedness as the default trajectory in the absence of competing forces. Future theoretical accounts of the formation of connectedness should consider whether connectedness simply reflects another form of either self-expansion or fulfilment of the need for relatedness—and, therefore, can be accounted for by existing theories—or if there is something unique that is not captured by existing frameworks.

## Conclusion

The literature on the psychological antecedents to connectedness is in a good place. There are associations of which we can be confident, such as the association between contact with nature and connectedness and between mindfulness and connectedness. There are, however, clear directions for future research. The priority should be placed on developing theories that help one understand the process through which known effects occur as well as on differentiating between different facets or types of a particular class of antecedents to better account for the heterogeneity identified in several of the antecedents. As the literature on the antecedents to connectedness continues to grow, and theories emerge, we will be better situated to leverage connectedness as a means of creating a more sustainably inclined society.

## Author Contributions

ML reviewed the literature and wrote the manuscript. JS provided input on the framing, organization, and presentation of the information in the manuscript as well as edits. All authors contributed to the article and approved the submitted version.

## Conflict of Interest

The authors declare that the research was conducted in the absence of any commercial or financial relationships that could be construed as a potential conflict of interest.

## Publisher’s Note

All claims expressed in this article are solely those of the authors and do not necessarily represent those of their affiliated organizations, or those of the publisher, the editors and the reviewers. Any product that may be evaluated in this article, or claim that may be made by its manufacturer, is not guaranteed or endorsed by the publisher.

## Footnotes

^1^This definition treats connectedness as distinct from what is most aptly referred to as environmental*ist* identity which instead focuses on whether one views themselves as a person who engages in various forms of pro-environmental behavior, or outright views themselves as an environmentalist (e.g., Kashima et al., 2014).

^2^The reason for this step was that many papers treating connectedness as a predictor include other variables as predictors within the same equation which taints our ability to draw conclusions about the association between the Y variable in the equation and connectedness to nature itself. The article pool was simply too large to allow us to read every single paper, therefore, we chose to exclude these papers since they had a high probability of being unusable for statistical reasons since this paper was focused on the antecedents of connectedness to nature.

^3^The discrepancy between these two findings is likely because in one study immersion was manipulated *via* a mental imagery script (Weinstein et al., 2009), whereas in the other immersion was manipulated in the form of the technology used (e.g., video vs. VR; Soliman et al., 2017).

^4^This effect was primarily true for individuals who held less positive environmental attitudes; individuals with highly positive environmental attitudes experienced similar levels of connectedness in either condition (Frantz et al., 2005) which may be reflective of a ceiling effect for connectedness among individuals who already hold strong proenvironmental attitudes.
